# Proliferation, Adhesion, and Morphology of Bone‐Derived Stromal Cells on Xenogenic Collagen Matrices: An In Vitro Study

**DOI:** 10.1002/cre2.70288

**Published:** 2026-01-08

**Authors:** Giulia Brunello, Direm Ilter, Florian Fürst, Jürgen Becker, Charlotte von Gall, Kathrin Becker, Beryl Schwarz‐Herzke

**Affiliations:** ^1^ Department of Oral Surgery University Hospital Düsseldorf Düsseldorf Germany; ^2^ Department of Orthodontics and Dentofacial Orthopaedics Charité—Universitätsmedizin Berlin Berlin Germany; ^3^ Institute of Anatomy II, Medical Faculty Heinrich Heine University Düsseldorf Germany

**Keywords:** cell viability, collagen membranes, cytoskeleton, protein expression, soft tissue augmentation

## Abstract

**Objectives:**

Acellular xenogeneic matrices are becoming increasingly popular for periodontal and peri‐implant soft tissue augmentation and recession coverage. This study aimed to investigate the behavior of bone‐derived stromal cells cultured onto three commercially available collagen membranes (Fibro‐Gide®, mucoderm®, and NovoMatrix®).

**Methods:**

Cells were isolated from alveolar bone chips of four adult healthy patients and were separately seeded on the membranes. Cells cultured in the absence of biomaterials were used as a control. Number and viability of cells on and around the membranes were evaluated at different timepoints. Actin cytoskeleton change was visualized with phalloidin staining. Markers for adhesion (VCAM‐1, FAK, and fibronectin) were assessed by immunofluorescence.

**Results:**

The number and viability of cells grown on the membranes were significantly lower than those of the controls at D7. Moreover, cells grown on and around all three membranes showed changes in actin cytoskeleton reminiscent of stress fibers. Cells grown around NovoMatrix® also show reduced viability. The three membranes had no effect on the adhesion markers of cells growing around them.

**Conclusions:**

All three membranes resulted in a reduction in viability and increased cell stress of adherent cells compared to the control. A reduced viability was detected, in particular, in cells growing around NovoMatrix®. Morphologically, the cells showed signs of stress. This reaction indicates that the membranes may release substances that could impair the healing process in vivo, especially in the case of fast degradation.

## 1 Introduction

Soft tissue grafting around teeth and implants plays a pivotal role in addressing several clinical scenarios, with indications spanning from periodontal and peri‐implant soft tissue augmentation to recession coverage (Deeb and Deeb 2015; Stefanini et al. 2023; Zucchelli and Mounssif 2015; Zucchelli et al. 2020).

Soft tissue augmentation can be achieved with subepithelial autogenous connective tissue grafts (CTGs), which have demonstrated predictable outcomes and long‐term stability (Barootchi et al. 2020; Carbone et al. 2024; Roccuzzo et al. 2024; Stefanini et al. 2023).

Soft tissue grafts can be harvested from different intraoral donor sites, including the palate, the edentulous ridges, the maxillary tuberosity, and the retromolar region. The palate is the preferred graft harvesting site, offering the highest tissue availability (Beymouri et al. 2023; D'Ambrosio et al. 2024; Zucchelli et al. 2020). Nevertheless, palatal harvesting has been associated with considerable patient discomfort in the donor area, especially in the case of grafts of bigger dimensions (Beymouri et al. 2023; D'Ambrosio et al. 2024; Zucchelli et al. 2014).

In order to reduce the pain at the donor site and accelerate tissue healing, materials like hyaluronic acid, hemostatic agents, concentrates, and protective dressings have been proposed (Gatti et al. 2023; Keceli et al. 2015; Puri et al. 2019; Scott et al. 2024). Moreover, subepithelial harvesting techniques have been introduced with the aim of obtaining primary intention healing and related reduced mobility (Zucchelli et al. 2020).

Nevertheless, to overcome the drawbacks associated with CTGs, soft tissue augmentation has recently witnessed a paradigm shift with the introduction of innovative xenogenic collagen soft tissue substitutes, offering the advantage of reduced surgical time, elimination of donor site morbidity, and enhanced patient acceptance (Dadlani et al. 2024; Montero et al. 2022; Thoma et al. 2023). Despite numerous clinical studies that have assessed the regeneration ability of these membranes (Montero et al. 2022; Tommasato et al. 2024), there is a scarcity of data reporting on the biological mechanisms underlying soft tissue healing and regeneration (Santos et al. 2023). To address this gap, we conducted an in vitro study in which bone‐derived stromal cells were cultured on commercially available acellular collagen membranes. To better replicate clinical conditions, cells isolated from human jawbone were preferred over cell lines, allowing for a more representative biological response (Czekanska et al. 2014; Harper 2024; Rothamel et al. 2004; Scheinpflug et al. 2018; Tanaka et al. 2022).

Therefore, the aim of this study was to investigate the viability, the actin cytoskeleton morphology, and markers for cell adhesion of stromal cells derived from bone of four donors grown on and/or around three different commercially available acellular collagen membranes for soft tissue augmentation.

## 2 Materials and Methods

This study was reported in accordance with the modified Consolidated Standards of Reporting Trials (CONSORT) guidelines for reporting in vitro studies on dental materials (Faggion 2012).

### 2.1 Xenogenic Collagen‐Based Matrices

The following commercially available collagen‐based membranes were utilized: Fibro‐Gide® (Geistlich Pharma AG, Wolhusen, Switzerland), mucoderm® (botiss biomaterials GmbH, Zossen, Germany), and NovoMarix® (CAMLOG Biotechnologies GmbH, Basel, Switzerland).

### 2.2 Isolation and Culture of Human Bone‐Derived Stromal Cells

Ethics approval was obtained from the Ethics Committee of the Medical Faculty of Heinrich Heine University, Düsseldorf (Protocol number: 2020‐1898_2). All participants were informed prior to the surgery and gave their written consent. Cells were isolated from bone chips harvested during routine dental implant placement from the jawbone of four healthy adult donors. The bone chips were cultured in nonbinding microplates (Corning® Costar®, Corning Inc., Corning, New York, the United States) in presence of Dulbecco's modified Eagle Medium high glucose (DMEM, Gibco, Thermo Fisher Scientific Inc., Waltham, Massachusetts, the United States), 20% Fetal Bovine Serum (FBS, Gibco), and 1% penicillin/streptomycin at 37°C and 5% CO_2_. Medium was changed every 2–3 days.

After early passages (3–5), 2000 cells were directly harvested (Day 0, D0) and seeded in 24‐well plates without a membrane (control) or containing one of the three collagen‐based membranes. The membranes were cut into square pieces of 5 mm × 5 mm, and the Fibro‐Gide® was horizontally split in half to obtain a height of 3 mm, similarly to the other membranes. Experiments were performed at different timepoints after seeding. For each experiment, two samples per patient, timepoint, and group were utilized.

### 2.3 Cell Viability

In order to evaluate cell viability, a luciferase‐based adenosine triphosphate (ATP) assay (CellTiter‐Glo® Assay, Promega GmbH, Walldorf, Germany) was used according to the manufacturer's instructions. First, the medium was aspirated and replaced with phosphate‐buffered saline (PBS). Then the membranes with the cells on them were transferred to a new well with PBS. Both the cells on the membranes and the cells that had grown in the original well in the presence of the membranes were further processed separately using the CellTiter‐Glo® buffers at Day 3 (D3) and Day 7 (D7). Viability of the cells without a membrane at D0, D3, and D7 was used as a reference. As a blank correction, medium without cells was measured at all timepoints. The optical density (OD) of luciferase was measured using a luminometer at 590–610 nm (FLUOstar Omega; BMG Labtech, Ortenberg, Germany) in duplicates.

### 2.4 Actin Cytoskeleton Organization

The actin cytoskeleton structure of cells grown on and around the membranes or without a membrane (control), cultured for 13 days, was analyzed using Phalloidin‐iFluor 488 Reagent (ab176753, Abcam, Cambridge, the United Kingdom), a phalloidin conjugate that binds to actin filaments (F‐actin), according to the manufacturer's instructions. Cells grown in the absence of membranes were used as a control. Briefly, the medium was aspirated, and the specimens were gently washed twice with PBS, followed by fixation with 4% formalin for 20 min at RT. Then the specimens were washed twice with PBS and exposed to 0.1% Triton X for 5 min. After rinsing twice with PBS, the supernatant was completely aspirated, replaced by 300 μL of Phalloidin conjugate working solution, and incubated at RT for 30 min. Counterstaining of cell nuclei was performed using DAPI (SouthernBiotech, Biozol, Eching, Germany), and samples were covered with coverslips using Fluoromount‐G® (Thermo Fischer Scientific).

### 2.5 Immunofluorescence

Cells grown on and around the membranes or without a membrane (control) for 7 or 14 days were analyzed by immunofluorescence to detect the vascular cell adhesion protein 1 (VCAM‐1), the focal adhesion kinase (FAK), and fibronectin (FN). Briefly, the medium was removed, each specimen was rinsed three times with PBS, and fixed for 10 min with 4% formalin (Sigma‐Aldrich, Merck, St. Louis, Missouri, the United States). Then the specimens were washed three times with PBS and stored at 4°C. PBS was replaced with a blocking buffer composed of 5% goat serum (Sigma‐Aldrich), 1% bovine albumin (BSA, Sigma‐Aldrich), and PBS with Triton X‐100 (Sigma‐Aldrich) and incubated for 1 h at RT. Subsequently, the cells were incubated with primary antibodies overnight at 4°C. The following antibodies were used: rabbit anti‐VCAM1 (1:1000, ab124047, Abcam) or rabbit anti‐FAK (1:100, CAB11195, AntibodyGenie, Biomol GmbH, Hamburg, Germany) with mouse anti‐FN (ab281574, Abcam). Subsequently, each specimen was rinsed three times for 15 min each with PBS and 0.1% Tween20 (Sigma‐Aldrich) followed by an incubation with secondary antibodies anti‐rabbit Alexa Fluor® 488 (1:5000; Abcam) and anti‐mouse Alexa Fluor® 569 (1:5000, Abcam) in a buffer containing 5% goat serum, 1% BSA, and PBS for 1 h at RT. The specimens were rinsed three times for 5 min each with PBS and 0.1% Tween20. The cell nuclei were stained with DAPI (SouthernBiotech, Biozol, Eching, Germany), and samples were covered with coverslips using Fluoromount‐G® (Thermo Fischer Scientific).

### 2.6 Fluorescence Microscopy

Fluorescence microscopic image stacks were captured using the KEYENCE BZ‐X800 microscope (Keyence Deutschland GmbH, Neu‐Isenburg, Germany). The microscope settings were optimized for each fluorophore and then maintained throughout image acquisition. Phalloidin staining was evaluated qualitatively. Immunofluorescence images were exported to ImageJ software (powered by Fiji), and cell count on the membranes, around the membranes, and on the control was performed over an area of 1500 µm × 1100 µm. In addition, the fluorescence signal of each marker in cells around the membranes (i.e., VCAM‐1, FAK, and FN) was evaluated by integrated density (IntDen = area (µm^2^) * mean gray value).

Details of cell counting and exemplarily quantification of VCAM‐1 immunofluorescence are provided in Figures S1 and S2, respectively.

### 2.7 Statistical Analysis

The R software (R Core Team 2024) was used for the statistical analysis. Boxplots with logarithmic scales were created for descriptive purposes. Linear mixed‐effects models were used for assessing the effect on cell viability of different membranes, separately for cells adhering to the membranes (on the membranes) and for cells around the membranes (fixed effects). Patients were considered as random factors, since data derived from cells from the same patient were considered dependent. At D0, the values were measured just in the absence of membranes, assuming no influence on cell viability of the membrane immediately after soaking; therefore, the control values at D0 were entered for all groups. For immunofluorescence analyses, linear mixed‐effects models were used as well. The interactions of timepoints and membranes were entered as fixed effects. For random effects, intercepts for patients were defined. For both analyses, in case of significance, pairwise post hoc multiple comparisons test using emmeans was performed with Tukey correction, and adjusted *p* values were reported. Visual inspection of residual and Q–Q plots did not reveal any obvious deviations from homoscedasticity or normality. The results were considered significant at *p* < 0.05.

## 3 Results

### 3.1 Effect of Membranes on Cell Number

**Figure 1:**
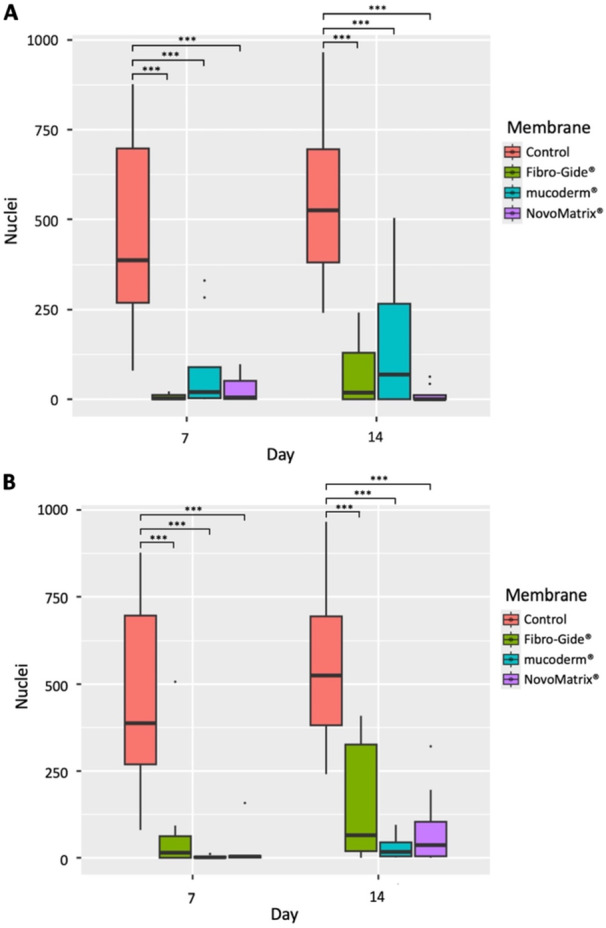
Effect of membrane on cell number. The number of DAPI‐stained cell nuclei was analyzed after cultivation in the absence (control) or grown on (A) and around (B) the respective membranes for 7 or 14 days on an area of 1500 µm × 1100 µm. *p < 0.05, **p < 0.01, ***p < 0.001. Since all groups were seeded with the same number of cells, and cell adhesion and marker expression require time to occur, D0 values were not included in the analysis of cell number and adhesion markers.

When counted on one of the three membranes for 7 or 14 days, significantly fewer cell nuclei were found than in the corresponding control (Figure 1A). Significant differences in the number of DAPI‐stained cell nuclei were observed between the groups (*p* < 0.001), with post hoc test confirming a significantly higher number of DAPI‐labeled nuclei in the control as compared to the membranes (Table S1). Similarly, when comparing the number of DAPI‐stained nuclei around the three membranes and in the control group (Figure 1B), significant differences were found between the groups (*p* < 0.001). The post hoc test confirmed that the number of DAPI‐labeled nuclei was significantly higher in the control group compared to the membranes, but no significant differences were observed between the membranes themselves. A trend toward a higher number of cells was observed at both timepoints on Fibro‐Gide®, although this difference was not statistically significant (Table S2).

### 3.2 Effect of Membranes on Cell Viability

**Figure 2:**
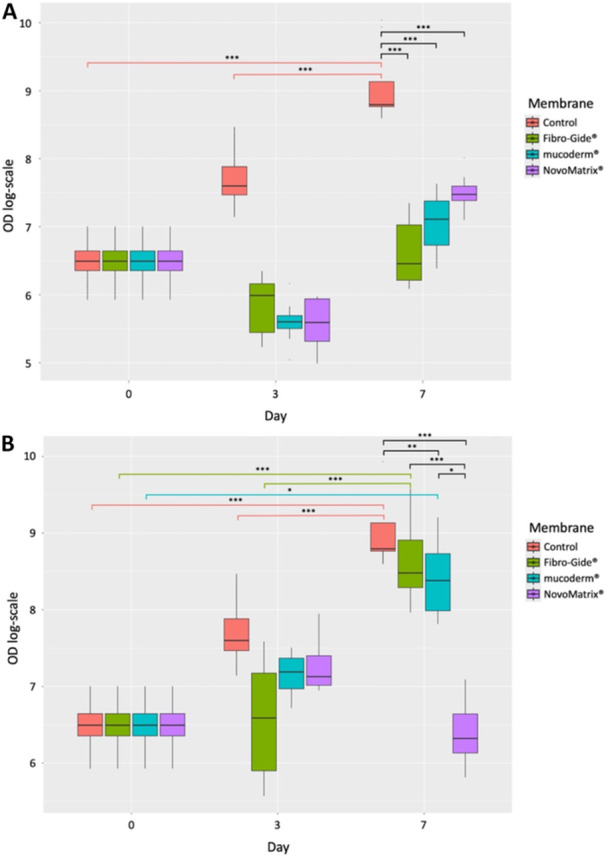
Effect of membranes on cell viability. Metabolically active, that is, viable cells were analyzed by an ATP‐based luciferase assay and expressed as optical density (OD). The cells were harvested after cultivation in the absence (control) or presence of the respective membranes for 3 or 7 days. (A) Viability of cells in the absence of a membrane (control) or grown on the membranes; (B) Viability of cells in the absence of a membrane (control) or that have grown around a membrane. *p < 0.05, **p < 0.01, ***p < 0.001.

The results of cell viability are presented in Figure 2. Mixed linear models confirmed significant differences among the groups for cells grown on the membranes (*p* < 0.001) and around the membranes (*p* < 0.001). Post hoc test was performed, and the adjusted *p* values from the post hoc test are reported in Tables S3 and S4.

In the control group, the cell viability significantly increased over time (Figure 2). In contrast, the viability of the cells grown on the membranes did not increase (Figure 2A). After 7 days, the viability of the cells grown on each of the three membranes is significantly lower than that of the control cells (Figure 2A).

However, there are differences in the effect on the viability of cells that have grown around the different membranes (Figure 2B). Cells grown around Fibro‐Gide® and Mucoderm® show a significant increase in viability over time (Figure 2B). In contrast, the viability of the cells grown around NovoMatrix® for 7 days is significantly lower than that of the control cells (Figure 2B). This indicates that this membrane affects the cell viability of cells present in the wells.

### 3.3 Effect of Membranes on Adhesion

**Figure 3:**
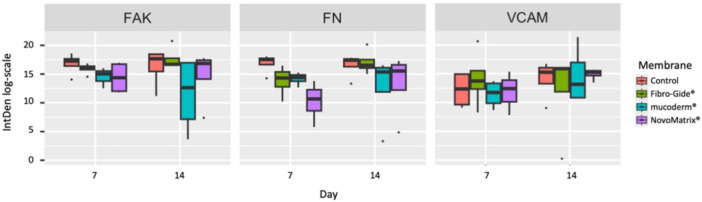
Effect of membranes on immunofluorescence against markers for cell adhesion. The intensity of immunofluorescence (IntDen) of markers for cell adhesion, such as focal adhesion kinase (FAK), fibronectin (FN), and vascular cell adhesion molecule (VCAM), in cells after cultivation in the absence (control) or grown around the respective membranes for 7 or 14 days. Since all groups were seeded with the same number of cells, and cell adhesion and marker expression require time to occur, D0 values were not included in the analysis of cell number and adhesion markers.

There was no significant difference in the intensity of immunofluorescence using markers for cell adhesion such as VCAM (*p* = 0.40), FAK (*p* = 0.34), or FN (*p* = 0.30) (Figure 3) in cells growing around the membranes.

This suggests that the decreased number of cells growing around the membranes is not due to reduced cell or adhesion involving VCAM, FAK, or FN.

### 3.4 Effect of Membranes on Actin Cytoskeleton Organization

**Figure 4:**
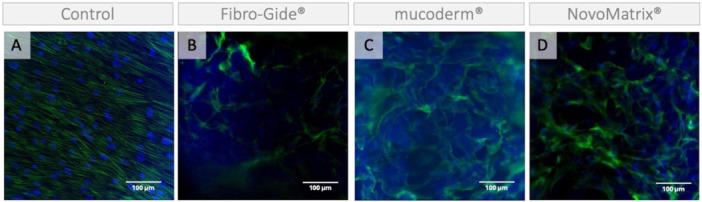
Actin cytoskeleton of cells growing on a membrane. Representative images of phalloidin fluorescence (green). Cells grown (A) in the absence of a membrane; on (B) Fibro‐Gide®, (C) mucoderm®, and (D) NovoMatrix® for 13 days. Cell nuclei are counterstained with DAPI (blue). Blurry blue staining is due to autofluorescence.

**Figure 5:**
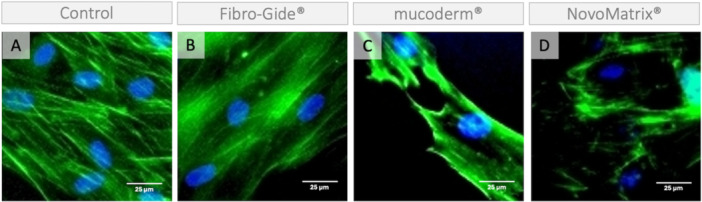
Actin cytoskeleton of cells growing around a membrane. Representative images of phalloidin fluorescence (green) of cells grown for 13 days (A) in the absence of a membrane; around (B) Fibro‐Gide®, (C) mucoderm®, and (D) NovoMatrix®. Cell nuclei are counterstained with DAPI (blue).

The images from control samples show fibroblast‐like spindle‐shaped cells with uniformly oriented actin filaments (Figures 4A and 5A). Cells growing *on* the membranes (Figure 4B–D) appear to be wrapped around the collagen fiber bundles and show reduced cell interconnections and unevenly distributed bundles of actin filaments, suggesting a cytoskeletal reorganization in response to the substrate. Representative images of cells growing *around* the membranes are presented in Figure 5. For example, cells growing around NovoMatrix® and mucoderm® show altered actin cytoskeleton organization reminiscent of stress fibers.

## 4 Discussion

The aim of our study was to investigate how the three different collagen membranes Fibro‐Gide®, mucoderm®, and NovoMatrix® affect bone‐derived stromal cells. These membranes, composed of various xenogeneic collagens, are designed for soft tissue augmentation and are supposed to enable optimal cell repopulation. Contrary to membranes for guided bone regeneration, whose occlusive properties prevent soft tissue colonization of the underlying bone defect during healing (Omar et al. 2019), membranes for soft tissue augmentation should maintain volume stability over time, while allowing the concomitant formation of new connective tissue and the gradual degradation and replacement of the matrices themselves (Vallecillo et al. 2021). Although the three membranes present the same main constituents, that is, collagen type I and III and elastin, Fibro‐Gide® is the only one presenting smart chemical cross‐linking and a highly porous structure (Vallecillo et al. 2021).

Based on the cell nuclei staining, significantly fewer cells were found on the membranes than in the control. Consistently, cells grown on any of the three membranes show reduced cell viability in the ATP assay. The reduced cell number could be due to reduced cell survival or to migration from the surface of the membranes to deeper parts, where we can no longer detect them with our method. Cells growing on the membranes show a change in the morphology of the actin cytoskeleton. Although the analysis was very limited due to the autofluorescence of the membranes (Neo et al. 2015), we noticed that the cells appeared to be wrapped around the collagen fiber bundles and showed fewer cell interconnections and unevenly distributed bundles of actin filaments, reminiscent of stress fibers (DesMarais et al. 2019; Han et al. 2022; Riedel et al. 2025). Fewer cell contacts indicate limited intercellular communication, which is crucial for cell survival (Han et al. 2022). Thus, our data suggest that the cells on the membranes have reduced viability due to cellular stress.

Since the effect on the surrounding tissue is crucial for healing after soft tissue augmentation and recession coverage, we investigated the effect on the cells that grow in the vicinity of the membrane. Cells growing near mucoderm® and NovoMatrix® showed significantly reduced viability. However, the cell viability assay used did not provide the possibility to distinguish gradient‐dependent effects on cell adherence to the well. The observed reduction in viability is likely a result of overall factors released from the materials, rather than proximity to the membrane itself. Therefore, these results should be interpreted with caution and not overextended beyond the general trend of reduced cell viability. Besides, alterations in the actin–cytoskeleton organization were observed, further suggesting that these membranes may release substances influencing cellular stress responses, warranting future investigation into their potential impact on cell viability. However, markers of cell adhesion, FAK, FN, and VCAM (Darribère and Schwarzbauer 2000; Murphy et al. 2020; Niibe et al. 2017), were not altered in cells growing near any of the membranes. This suggests that the membranes do not affect the cell–matrix interaction.

According to this study, to optimize the development of acellular xenogeneic matrices, special attention should be paid to reducing cell stress and promoting cell viability. Several factors could influence cell viability and trigger cellular stress, such as the chemical composition, structure, porosity, stiffness, and degradation pattern of the membrane (Solderer et al. 2023; Vallecillo et al. 2021). The in vitro model established in this study could be used in future studies, for example, to simulate the degradation kinetics that occur in vivo when collagen‐based membranes are exposed to the oral environment (Calciolari et al. 2018). Also, the effect of excessive or prolonged inflammation on membrane‐supported soft tissue regeneration could be investigated in this in vitro model by using pro‐inflammatory substances. Considering the clinical indications of these membranes (Montero et al. 2022; Tommasato et al. 2024), it might also be relevant to explore the potential of the membranes to induce differentiation of stem cells into fibroblasts and endothelial cells.

The visualization of cytoskeletal morphology and the quantification of different markers were hindered by autofluorescence (Neo et al. 2015), presenting a significant challenge in our analysis. This issue was particularly pronounced when examining the cytoskeleton and the expression of the markers of cells on the membrane, further complicating the interpretation of the results. Additionally, a key limitation of the study lies in the comparison between the 2D culture model and the 3D membrane environment. Reduced cell growth on 3D matrices might have been due to structural, mechanical, and diffusion‐related factors. Finally, a limitation of this study is the reduced number of donors, as inter‐donor variability may have influenced the observed membrane‐associated effects. In light of patient‐dependent variability in wound healing, future studies with a larger donor cohort could enable the identification of donor‐specific trends in cell proliferation, viability, and differentiation on the different membranes.

## 5 Conclusions

In conclusion, within the limitations of an in vitro study, contact with acellular xenogeneic matrices seems to induce cellular stress and reduced cell viability. Furthermore, two of the three membranes studied also exhibited a similar effect on cells grown nearby. Despite current evidence showing that these membranes are nonetheless well‐suited for soft tissue augmentation, they might release substances that could delay the healing process in vivo, especially in circumstances that promote faster degradation. Future studies should be addressed to clarify the degradation patterns of these membranes and data confirmed in vivo.

## Author Contributions

Conceptualization: G.B., J.B., K.B., and B.S.H. Methodology: G.B. and B.S.H. Validation: B.S.H. Formal analysis: D.I., F.F., and B.S.H. Investigation: D.I., F.F., and B.S.H. Resources: G.B., J.B., and B.S.H. Data curation: G.B., C.V.G., K.B., and B.S.H. Writing – original draft: G.B. and B.S.H. Writing – review and editing: G.B., D.I., F.F., J.B., C.V.G., K.B., and B.S.H. Visualization: D.I. and F.F. Supervision: G.B., J.B., C.V.G., K.B., and B.S.H. Project administration: B.S.H.

## Ethics Statement

Ethics Committee of the Medical Faculty of Heinrich Heine University, Düsseldorf (Protocol number: 2020‐1898_2).

## Conflicts of Interest

The authors declare no conflicts of interest.

## Supporting information


**Supporting Figure 1:** Analysis of DAPI‐stained cell nuclei. **Supporting Figure 2:** Analysis of immunofluorescence.

**Supporting Table 1:** Cell nuclei labelled by DAPI on membranes and in control group. Adjusted p‐values from the post hoc test are reported. **Supporting Table 2:** Cell nuclei labelled by DAPI around membranes and in control group. Adjusted p‐values from the post hoc test are reported. **Supporting Table 3:** Viability of cells on the membranes. Adjusted p‐values from the post hoc test are reported. **Supporting Table 4:** Viability of cells around to the membranes. Adjusted p‐values from the post hoc test are reported.

## Data Availability

The data underlying this article will be shared on reasonable request to the corresponding author.
