# Supplementary material for: Proliferation, Adhesion, and Morphology of Bone‐Derived Stromal Cells on Xenogenic Collagen Matrices: An In Vitro Study
Source: Clin Exp Dent Res. 2026 Jan 8;12(1):e70288. doi: 10.1002/cre2.70288 (PMC12784116; doi:10.1002/cre2.70288)
Supplement: Supplementary file 1 — Supporting Figure 1: Analysis of DAPI‐stained cell nuclei. Supporting Figure 2: Analysis of immunofluorescence. [file CRE2-12-e70288-s002.docx]

**SUPPLEMENTARY FIGURES**


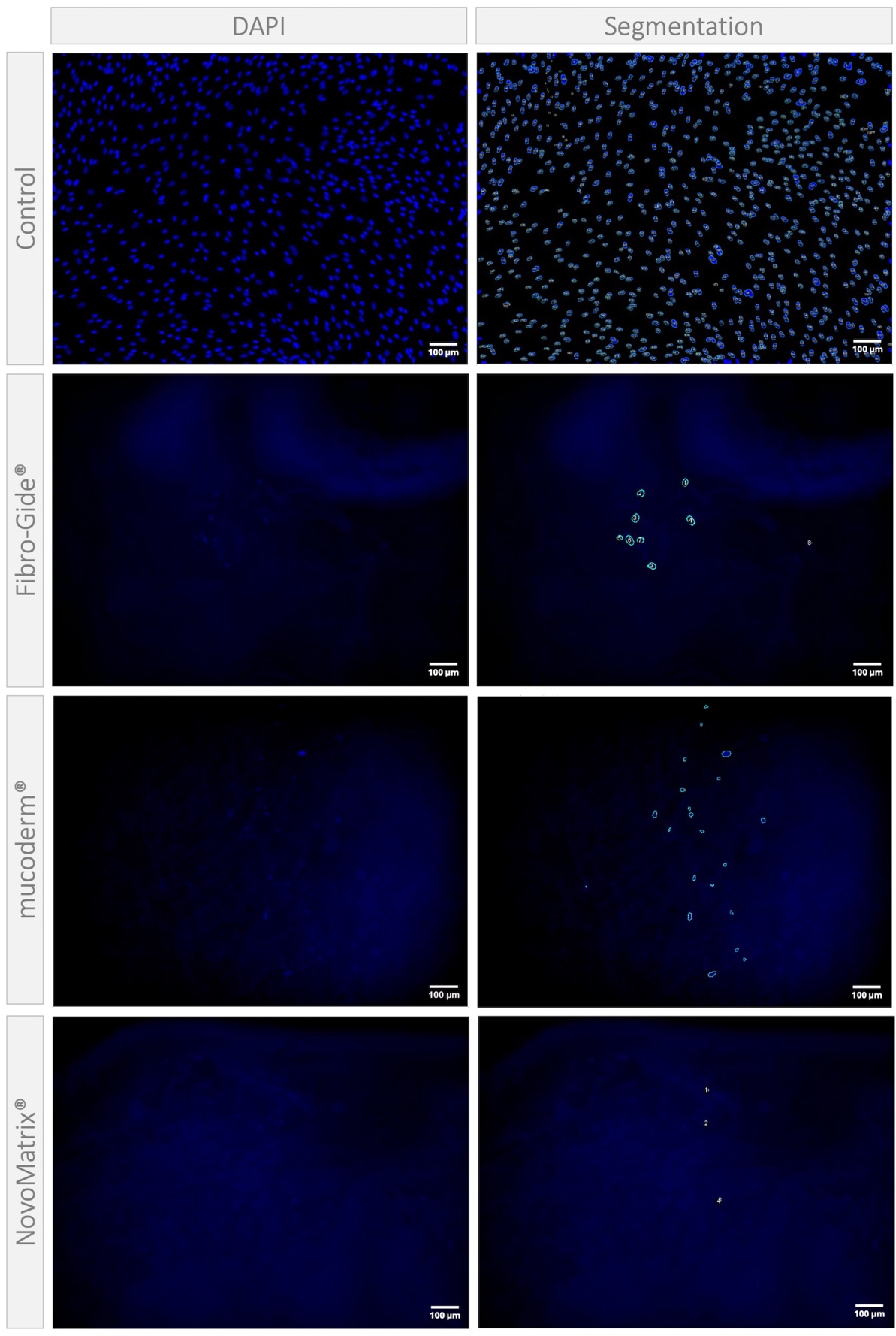


**Suppl. Figure 1.** **Analysis of DAPI-stained cell nuclei**

Representative images of DAPI-stained cell nuclei in control and on the three membranes (i.e. Fibro-Gide®, mucoderm® and NovoMatrix®) and related semi-automatic segmentation for quantification using ImageJ on an area of 1500µm x 1100µm.

**
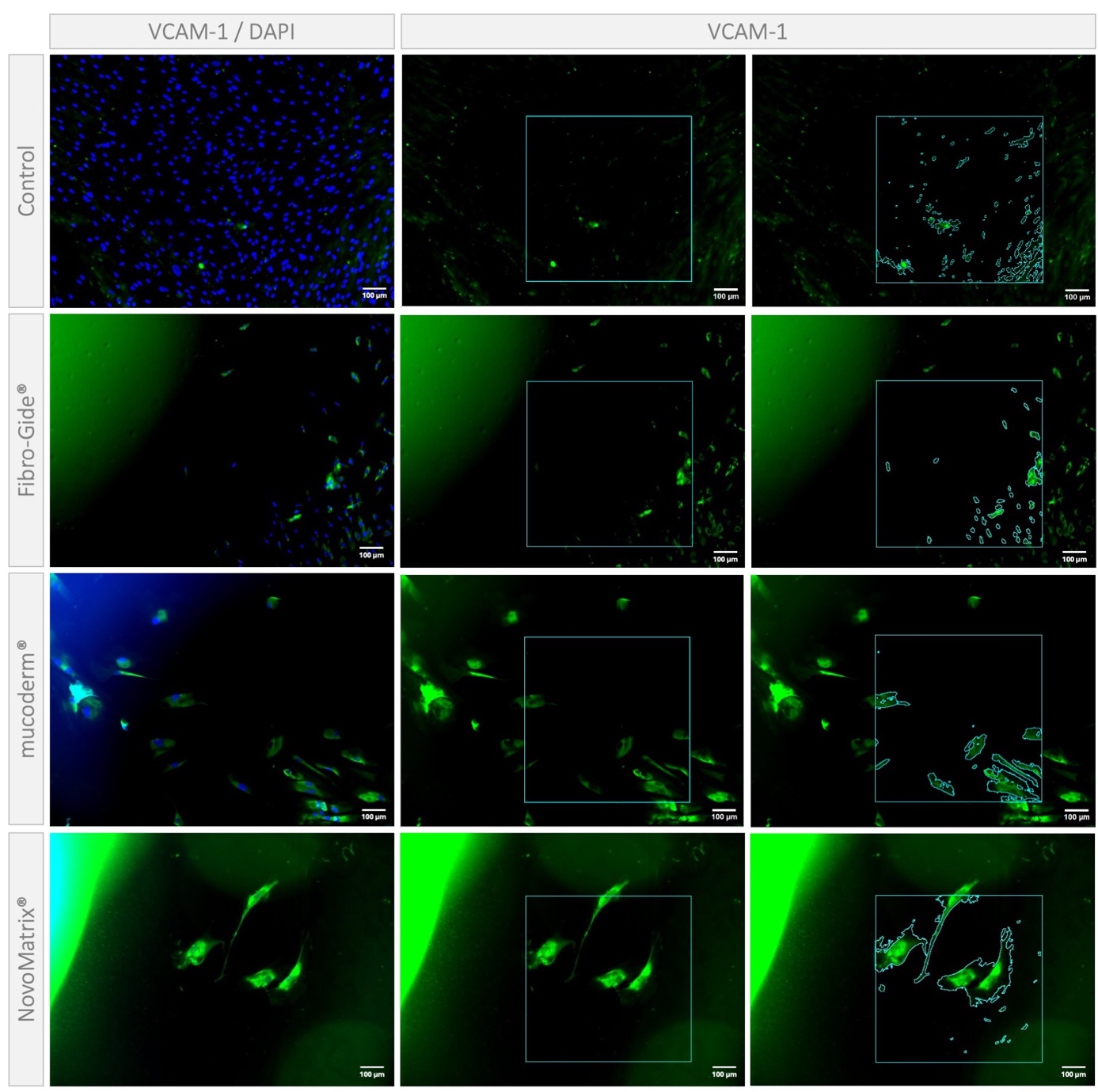
**

**Suppl. Figure 2. Analysis of immunofluorescence**

Representative immunofluorescence of cells in the control or grown around the three membranes (i.e. Fibro-Gide®, mucoderm® and NovoMatrix®) for VCAM-1 (green)/DAPI (blue), and related semi-automatic segmentation for quantification using ImageJ. Fluorescence intensity above background was calculated in randomly selected square areas of 700µm x 700µm area.
