# Supplementary material for: Proliferation, Adhesion, and Morphology of Bone‐Derived Stromal Cells on Xenogenic Collagen Matrices: An In Vitro Study
Source: Clin Exp Dent Res. 2026 Jan 8;12(1):e70288. doi: 10.1002/cre2.70288 (PMC12784116; doi:10.1002/cre2.70288)
Supplement: Supplementary file 2 — Supporting Table 1: Cell nuclei labelled by DAPI on membranes and in control group. Adjusted p‐values from the post hoc test are reported. Supporting Table 2: Cell nuclei labelled by DAPI around membranes and in control group. Adjusted p‐values from the post hoc test are reported. Supporting Table 3: Viability of cells on the membranes. Adjusted p‐values from the post hoc test are reported. Supporting Table 4: Viability of cells around to the membranes. Adjusted p‐values from the post hoc test are reported. [file CRE2-12-e70288-s001.docx]

**SUPPLEMENTARY TABLES**

**Suppl. Table 1.** Cell nuclei labelled by DAPI on membranes and in control group. Adjusted p-values from the post hoc test are reported.

**A**

| **Grouping variable** | **Comparator 1** | **Comparator 2** | **p-value** |
| --- | --- | --- | --- |
| D7 | Control | Fibro-Gide® | **< 0.001***** |
|  | Control | mucoderm® | **< 0.001***** |
|  | Control | NovoMatrix® | **< 0.001***** |
|  | Fibro-Gide® | mucoderm® | 0.93 |
|  | Fibro-Gide® | NovoMatrix® | 1.00 |
|  | mucoderm® | NovoMatrix® | 0.99 |
| D14 | Control | Fibro-Gide® | **< 0.001***** |
|  | Control | mucoderm® | **< 0.001***** |
|  | Control | NovoMatrix® | **< 0.001***** |
|  | Fibro-Gide® | mucoderm® | 0.87 |
|  | Fibro-Gide® | NovoMatrix® | 0.99 |
|  | mucoderm® | NovoMatrix® | 0.34 |
| Control | D7 | D14 | 0.91 |
| Fibro-Gide® | D7 | D14 | 0.98 |
| mucoderm® | D7 | D14 | 0.95 |
| NovoMatrix® | D7 | D14 | 1.00 |

Significant values are labelled: * p < 0.05, ** p< 0.01, *** p < 0.001.

**Suppl. Table 2.** Cell nuclei labelled by DAPI around membranes and in control group. Adjusted p-values from the post hoc test are reported.

| **Grouping variable** | **Comparator 1** | **Comparator 2** | **p-value** |
| --- | --- | --- | --- |
| D7 | Control | Fibro-Gide® | **< 0.001***** |
|  | Control | mucoderm® | **< 0.001***** |
|  | Control | NovoMatrix® | **< 0.001***** |
|  | Fibro-Gide® | mucoderm® | 0.93 |
|  | Fibro-Gide® | NovoMatrix® | 0.98 |
|  | mucoderm® | NovoMatrix® | 1.00 |
| D14 | Control | Fibro-Gide® | **< 0.001***** |
|  | Control | mucoderm® | **< 0.001***** |
|  | Control | NovoMatrix® | **< 0.001***** |
|  | Fibro-Gide® | mucoderm® | 0.59 |
|  | Fibro-Gide® | NovoMatrix® | 0.96 |
|  | mucoderm® | NovoMatrix® | 0.99 |
| Control | D7 | D14 | 0.93 |
| Fibro-Gide® | D7 | D14 | 0.96 |
| mucoderm® | D7 | D14 | 0.99 |
| NovoMatrix® | D7 | D14 | 0.99 |

Significant values are labelled: * p < 0.05, ** p< 0.01, *** p < 0.001.

**Suppl. Table 3.** Viability of cells on the membranes. Adjusted p-values from the post hoc test are reported.

| **Grouping variable** | **Comparator 1** | **Comparator 2** | **p-value** |
| --- | --- | --- | --- |
| D0 | Control | Fibro-Gide® | 1.00 |
|  | Control | mucoderm® | 1.00 |
|  | Control | NovoMatrix® | 1.00 |
|  | Fibro-Gide® | mucoderm® | 1.00 |
|  | Fibro-Gide® | NovoMatrix® | 1.00 |
|  | mucoderm® | NovoMatrix® | 1.00 |
| D3 | Control | Fibro-Gide® | 0.63 |
|  | Control | mucoderm® | 0.57 |
|  | Control | NovoMatrix® | 0.57 |
|  | Fibro-Gide® | mucoderm® | 1.00 |
|  | Fibro-Gide® | NovoMatrix® | 1.00 |
|  | mucoderm® | NovoMatrix® | 1.00 |
| D7 | Control | Fibro-Gide® | **< 0.001***** |
|  | Control | mucoderm® | **< 0.001***** |
|  | Control | NovoMatrix® | **< 0.001***** |
|  | Fibro-Gide® | mucoderm® | 1.00 |
|  | Fibro-Gide® | NovoMatrix® | 1.00 |
|  | mucoderm® | NovoMatrix® | 1.00 |
| Control | D0 | D3 | 0.82 |
|  | D0 | D7 | **< 0.001***** |
|  | D3 | D7 | **< 0.001***** |
| Fibro-Gide® | D0 | D3 | 1.00 |
|  | D0 | D7 | 1.00 |
|  | D3 | D7 | 1.00 |
| mucoderm® | D0 | D3 | 1.00 |
|  | D0 | D7 | 1.00 |
|  | D3 | D7 | 1.00 |
| NovoMatrix® | D0 | D3 | 1.00 |
|  | D0 | D7 | 0.99 |
|  | D3 | D7 | 0.91 |

Significant values are labelled: * p < 0.05, ** p< 0.01, *** p < 0.001.

**Suppl. Table 4.** Viability of cells around to the membranes. Adjusted p-values from the post hoc test are reported.

| **Grouping variable** | **Comparator 1** | **Comparator 2** | **p-value** |
| --- | --- | --- | --- |
| D0 | Control | Fibro-Gide® | 1.00 |
|  | Control | mucoderm® | 1.00 |
|  | Control | NovoMatrix® | 1.00 |
|  | Fibro-Gide® | mucoderm® | 1.00 |
|  | Fibro-Gide® | NovoMatrix® | 1.00 |
|  | mucoderm® | NovoMatrix® | 1.00 |
| D3 | Control | Fibro-Gide® | 0.98 |
|  | Control | mucoderm® | 1.00 |
|  | Control | NovoMatrix® | 1.00 |
|  | Fibro-Gide® | mucoderm® | 1.00 |
|  | Fibro-Gide® | NovoMatrix® | 1.00 |
|  | mucoderm® | NovoMatrix® | 1.00 |
| D7 | Control | Fibro-Gide® | 0.21 |
|  | Control | mucoderm® | **< 0.01**** |
|  | Control | NovoMatrix® | **< 0.001***** |
|  | Fibro-Gide® | mucoderm® | 0.99 |
|  | Fibro-Gide® | NovoMatrix® | **< 0.001***** |
|  | mucoderm® | NovoMatrix® | **0.02*** |
| Control | D0 | D3 | 0.96 |
|  | D0 | D7 | **< 0.001***** |
|  | D3 | D7 | **< 0.001***** |
| Fibro-Gide® | D0 | D3 | 1.00 |
|  | D0 | D7 | **< 0.001***** |
|  | D3 | D7 | **< 0.001***** |
| mucoderm® | D0 | D3 | 1.00 |
|  | D0 | D7 | **0.03*** |
|  | D3 | D7 | 0.11 |
| NovoMatrix® | D0 | D3 | 1.00 |
|  | D0 | D7 | 1.00 |
|  | D3 | D7 | 1.00 |

Significant values are labelled: * p < 0.05, ** p< 0.01, *** p < 0.001.
